# Insights into the SAM Synthetase Gene Family and Its Roles in Tomato Seedlings under Abiotic Stresses and Hormone Treatments

**DOI:** 10.3390/plants9050586

**Published:** 2020-05-04

**Authors:** Parviz Heidari, Faezeeh Mazloomi, Thomas Nussbaumer, Gianni Barcaccia

**Affiliations:** 1 Faculty of Agriculture, Shahrood University of Technology, Shahrood 3619995161, Iran; faezeh_mazloomi@yahoo.com; 2 Helmholtz Zentrum München, Deutsches Forschungszentrum für Gesundheit und Umwelt (GmbH), 85764 Neuherberg, Germany; thomasnbionf@gmail.com; 3 Laboratory of Genomics for Breeding, DAFNAE, University of Padova, Campus of Agripolis, 35030 Legnaro, Italy; gianni.barcaccia@unipd.it

**Keywords:** genome analysis, post translation modifications, miRNAs target site, hormones, abiotic stress

## Abstract

S-Adenosyl-L-methionine (SAM) is a key enzyme involved in many important biological processes, such as ethylene and polyamine biosynthesis, transmethylation, and transsulfuration. Here, the SAM synthetase (SAMS) gene family was studied in ten different plants (Arabidopsis, tomato, eggplant, sunflower, *Medicago truncatula*, soybean, rice, barley, *Triticum urartu* and sorghum) with respect to its physical structure, physicochemical characteristics, and post-transcriptional and post-translational modifications. Additionally, the expression patterns of SAMS genes in tomato were analyzed based on a real-time quantitative PCR assay and an analysis of a public expression dataset. SAMS genes of monocots were more conserved according to the results of a phylogenetic analysis and the prediction of phosphorylation and glycosylation patterns. SAMS genes showed differential expression in response to abiotic stresses and exogenous hormone treatments. Solyc01g101060 was especially expressed in fruit and root tissues, while Solyc09g008280 was expressed in leaves. Additionally, our results revealed that exogenous BR and ABA treatments strongly reduced the expression of tomato SAMS genes. Our research provides new insights and clues about the role of SAMS genes. In particular, these results can inform future functional analyses aimed at revealing the molecular mechanisms underlying the functions of SAMS genes in plants.

## 1. Introduction

**Figure 1:**
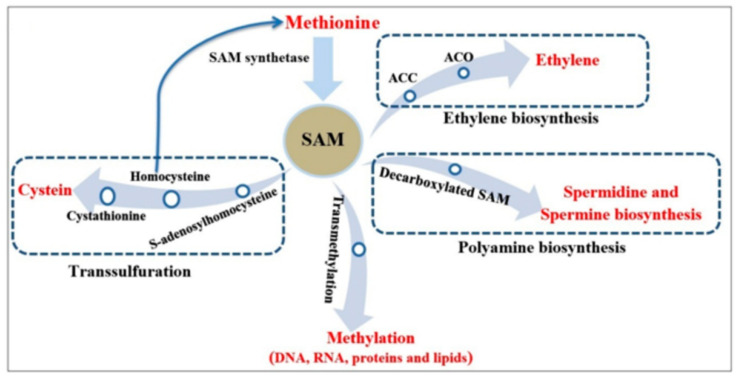
Metabolic and biosynthesis pathways involving SAM.

S-Adenosyl-L-methionine (SAM) is a key molecule in eukaryotic cells that participates in several important biological reactions [1,2,3]. SAM is catalyzed from methionine and adenosine triphosphate (ATP) by the enzyme S-adenosylmethionine synthetase (SAMS) [3]. SAM is involved in different pathways, such as ethylene (in plants), nicotianamine and polyamine biosynthesis pathways; transmethylation and transsulfuration pathways, and the methionine metabolism pathway [4,5] (Figure 1). In addition, SAM participates in lignin biosynthesis and responses to environmental stresses, and it has essential roles in several cellular processes [6,7]. SAM is widely known as a methyl group donor, with many proteins, nucleic acids, phospholipids, and small molecules being methylated mainly by SAM [8]. SAM, as a sulfonium salt, has a sulfur atom in its structure that is involved in transferring the methyl group to nucleophilic atoms such as carbon atom (in C5-cytosine of DNA strands), nitrogen atom (in amino group of glycine), sulfur atom (in S-methylation reaction of thiopurines), oxygen atom (in O-methylation reaction of catecholamine) and arsenic atom [9,10]. Furthermore, aminopropyl groups involved in polyamine and ethylene biosynthesis and methylene groups involved in cyclopropyl fatty acid biosynthesis use SAM as a methyl donor source [3].

In the transsulfuration pathway, SAM enzyme participates in the conversion of homocysteine to cysteine as a precursor of cellular antioxidants such as glutathione [1]. After methylation, SAM is converted to S-adenosylhomocysteine (SAH), which is a vigorous inhibitor of methylation reactions. S-adenosylhomocysteine hydrolase enzyme converts SAH to homocysteine and adenosyl [11]. Recent studies have shown that several human diseases, such as stroke, dementia, osteoporosis, Alzheimer disease and coronary artery disease, are correlated with plasma total homocysteine [11,12,13]. Furthermore, SAM is a substrate for polyamine and ethylene biosynthesis. Polyamines are important hormones in eukaryote organisms and regulate cell growth during several activities, including stress responses, pollen and flower development and protection of photosystem II [1,5,14]. In polyamine biosynthesis, SAM is converted to S-adenosylmethioninamine enzyme (also known as decarboxylated SAM) by S-adenosylmethionine decarboxylase (SAMDC) [5]. S-adenosylmethioninamine, as an aminopropyl donor, is used in the biosynthesis of polyamines, such as spermine and spermidine from putrescine (Figure 1) [5]. In addition, SAM participates in the synthesis of 1-aminocyclopropane-1-carboxylic acid (ACC) by ACC synthase (ACS); ACC, as a precursor, is converted to ethylene by ACC oxidase (ACO) [15,16]. Previous studies show that genes involved in the SAM cycle (Figure 1) play important roles in regulating cell growth and stress responses. For instance, the downregulation of SAMDC genes reduces pollen viability, plant length and abiotic stress tolerance in transgenic rice [17]. In addition, overexpression of SAMDC increases drought tolerance in Arabidopsis [18], sodium chloride-stress tolerance in rice [19], and biotic and abiotic stress tolerance in tomato [20].

SAM synthetase (SAMS) enzyme, also known as methionine adenosyltransferase (MAT), catalyzes SAM from methionine. Espartero et al. [21] found that SAMS genes of tomato have differential expression in response to salt stress. In addition, Li et al. [22] observed that overexpression of the SAM synthetase gene of *Lycoris radiate* in *E. coli* can promote plant tolerance to salt stress. Furthermore, Mao et al. [23] found that a plasma membrane receptor-like kinase, FERONIA (FER), negatively regulates the synthesis of SAM by SAMS in Arabidopsis and that ethylene and SAM content are increased in a *fer* mutant. Interestingly, Erb et al. [24] detected an unexpected link between SAM metabolism and RubisCO-like protein in the central carbon metabolism of *Rhodospirillum rubrum*.

The above-mentioned observations indicate that the SAMS gene family plays critical roles in the metabolism of plant species and is involved in regulating cell growth and gene expression. Several studies have examined the roles of SAMS genes in human diseases, but comparative studies of the structure, physicochemical properties, and expression of SAMS gene family in plants are lacking. In this study, we conducted a comparative investigation of SAMS genes in dicots (Arabidopsis, tomato, eggplant, sunflower, *Medicago truncatula*, and soybean) and monocots (rice, barley, *Triticum urartu* and sorghum), with focus on their structure, physicochemical characteristics and post-transcriptional and post-translational modifications (miRNA targets, phosphorylation, and glycosylation). Additionally, using RNA-seq data, we studied the gene expression of SAMS genes under different treatments in tomato. Furthermore, the gene expression patterns of tomato-SAMS genes were investigated under abiotic stresses (salt, cold, heat, and water stress) and hormone treatments (brassinosteroid, salicylic acid, auxin, and abscisic acid) using real-time quantitative PCR (RT-qPCR) assay. This study yields important information about SAMS genes that can inform future studies of the functions of SAMS genes.

## 2. Results

### 2.1. Physiochemical Properties of SAMS Genes

The physicochemical properties of the SAMS genes are shown in Table 1. The table lists gene ID, amino acid sequence length, molecular weight, isoelectric point, instability index, aliphatic index, GRAVY value, and subcellular localization. In our study, the number of SAMS genes in the selected plant species varied from 3 to 9, and the highest copy number was observed in soybean. The length of the amino acid sequence ranged from 238 to 479, with HanXRQChr01g0027791 (sunflower SAMS) being the smallest protein and EMS52834.1 (*T. urartu* SAMS) being the largest protein. The molecular weight (MW) of the studied SAMS proteins ranged from 26.22 to 52.80 KDa. The isoelectric point (pI) varied from 5.22 to 9.13; LOC_Os01g18860 (rice SAMS) was predicted to be an acidophilic protein, while HanXRQChr01g0027791 was predicted to be an alkaline protein. According to the protein instability index values, all studied SAMS proteins were stable. The aliphatic index, a measure of thermostability, varied from 79.87 to 92.52. The predicted GRAVY value of the studied SAMS proteins ranged from −0.353 to −0.153, and most of the SAMS genes showed negative values, indicating hydrophilic properties. The prediction of subcellular localization showed that the most SAMS proteins were localized in the cytoplasm. However, most SAMS proteins of sunflowers were predicted to localize in the chloroplast (Table 1).

### 2.2. Phylogenetic and Conserved Motif Analyses of the SAMS Gene Family 

**Figure 2:**
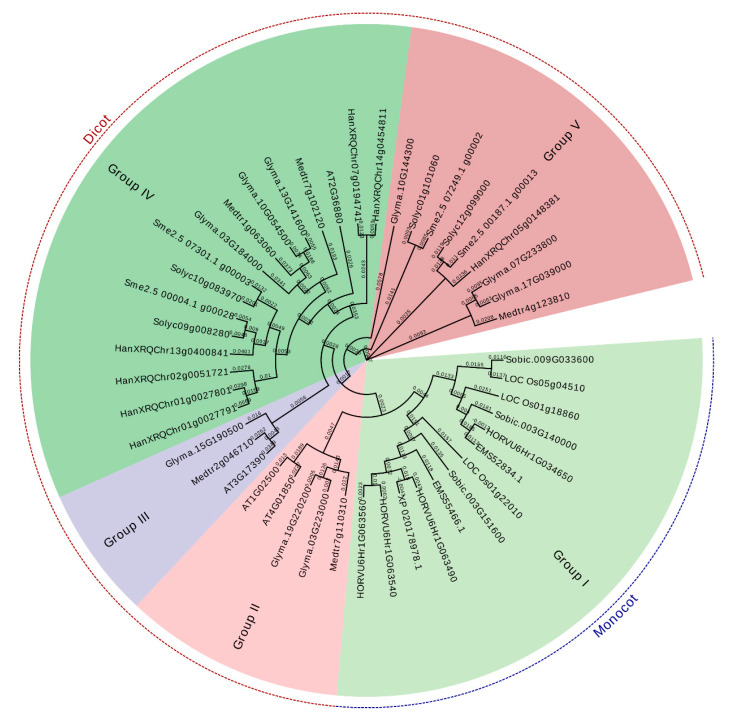
Phylogenic tree of SAMS gene-based amino acid sequences using the neighbor-joining method of Clustal Omega.

**Figure 3:**
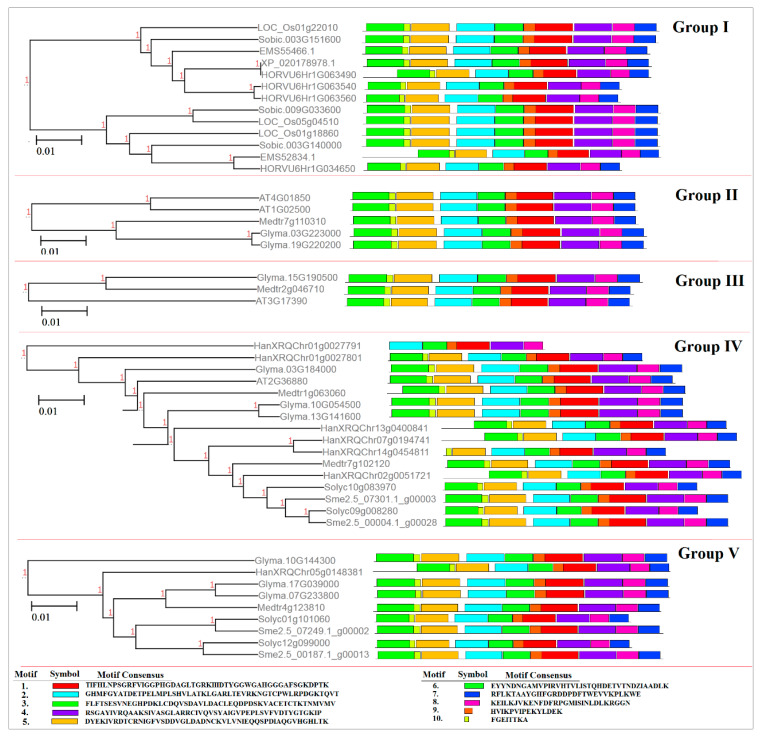
Conserved motif distribution of SAMS proteins in the studied plant species predicted using the MEME v4.12.0 server.

The evolutionary relationships of the SAMS genes were analyzed by the multiple alignment of the amino acid sequences from Arabidopsis, tomato, eggplant, sunflower, barley, rice, sorghum, *Medicago truncatula, Triticum urartu* and soybean using the neighbor-joining method (Figure 2). The phylogenetic analysis clustered the SAMS genes of the selected plant species into five major groups. All SAMS genes of monocots were located in the first group, while those of dicots were clustered into four different groups. In addition, the SAMS genes of group V showed more variability than the SAMS genes of the other groups (Figure 2). According to the phylogenetic analysis, the SAMS genes of soybean, *M. truncatula*, and Arabidopsis were more similar to each other than to the other SAMS genes. Moreover, we evaluated the conserved motifs according to the phylogenetic analysis to gain insight into the structural features of SAMS members in selected plants. The conserved motifs were predicted using the MEME server; 10 conserved motifs in the SAMS proteins were predicted (Figure 3). All members of the SAMS gene family contain motifs 1, 2, 3 and 8 (Figure 3). Determining the functions of these conserved motifs may provide insight into the functions and interactions of SAMS genes.

### 2.3. Prediction of Phosphorylation and Glycosylation Sites

**Figure 4:**
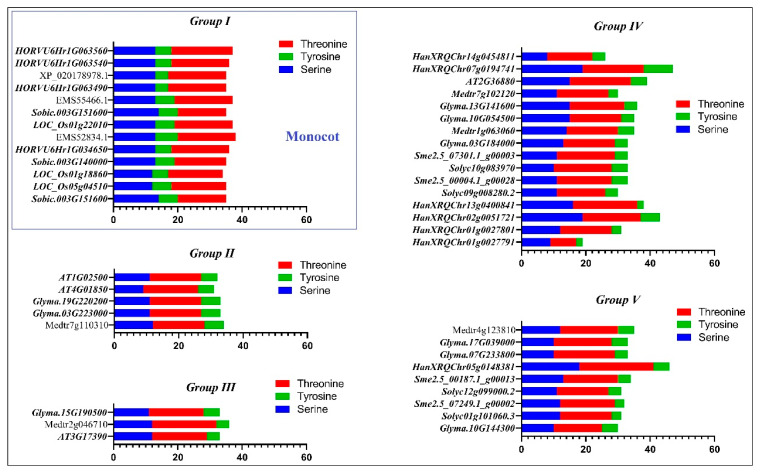
Predicted phosphorylation sites of SAMS proteins in different plant species determined using the NetPhos 3.1 server.

**Figure 5:**
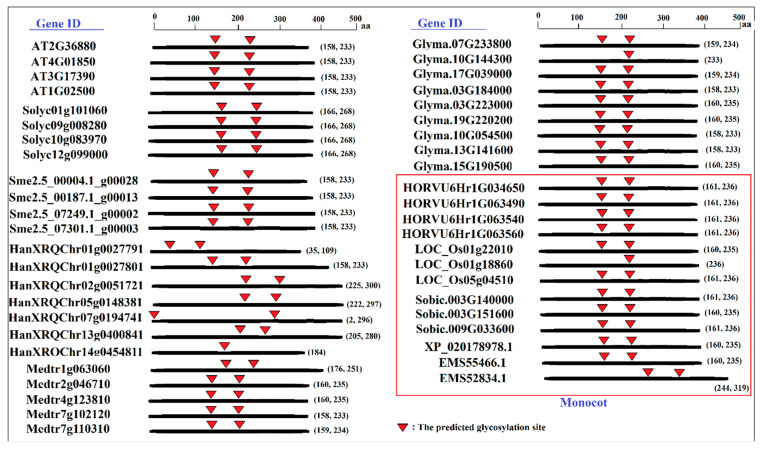
Predicted glycosylation sites of SAMS proteins in different plant species determined using the NetNGlyc 1.0 server.

In this study, the phosphorylation and glycosylation sites were predicted to provide insight into the post-translation modifications of SAMS proteins. The potential phosphorylation sites of SAMS proteins were predicted using the NetPhos 3.1 server (Figure 4). The number of predicted phosphorylation sites ranged from 19 in HanXRQChr01g0027791 to 47 in HanXRQchr07g0194741 (sunflower SAMS). The most studied SAMS proteins had 30 to 40 predicted phosphorylation sites, and most of the variation was observed among the SAMS proteins of sunflower. Among the studied SAMS proteins, three sunflower SAMS proteins (HanXRQChr07g0194741, HanXRQChr02g0051721 and HanXRQChr05g0148381) were identified as serine-rich proteins (Figure 4). The amino acid sequences of SAMS proteins were analyzed using the NetNGlyc 1.0 server to predict N-glycosylation sites. The prediction results revealed that most SAMS proteins were glycosylated at two conserved sites (Figure 5). The amino acid 235/236 was predicted to be a conserved glycosylation site in all studied SAMS proteins of monocots.

### 2.4. Prediction of MicroRNA Target Sites

**Figure 6:**
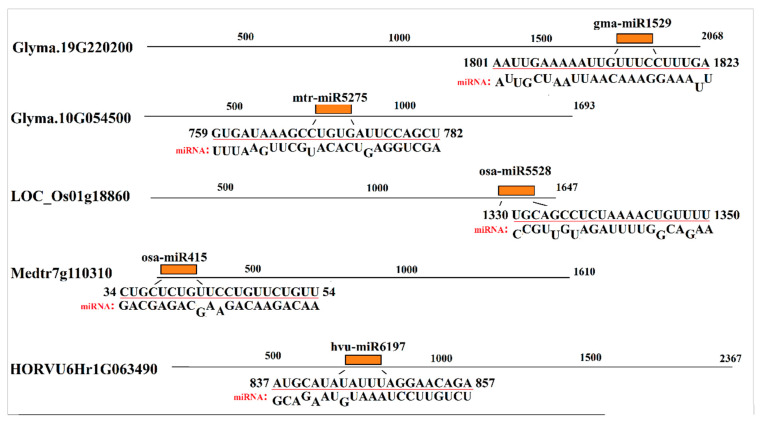
Predicted sites of microRNA targets in transcript sequences of the studied SAMS genes identified using the psRNATarget server.

MicroRNAs are noncoding RNAs that play critical roles in many biological processes. The transcript sequences of SAMS genes were screened to predict the target sites of plant microRNAs using the psRNATarget server. The prediction of miRNA-target sites revealed that transcripts of SAMS genes were targeted by plant miRNAs at an expectation level ≤ 2.5 (Figure 6). The binding sites of other miRNAs with lower complementarity (expectation level <4) were also predicted, as shown in Table S2. The binding sites of gma-miR1520 and mtr-miR5275 were observed in transcript sequences of two soybean SAMS genes, Glyma.19G220200 and Glyma.10G054500. Furthermore, the pre-mRNA of LOC_Os01g18860, a rice-SAMS gene, was targeted by osa-miR5528. In addition, the binding site of osa-miR415 was observed in transcript sequences of the Medtr7g110310 gene, and a barley SAMS gene (HORVU6Hr1G0634490) was predicted to be targeted by hvu-miR6197. According to our results, SAMS genes can be regulated by different miRNAs and that members of this complex (SAMS-miRNAs) can affect downstream pathways related to SAM.

### 2.5. Transcriptional Profiling of SAMS Genes in Tomato

**Figure 7:**
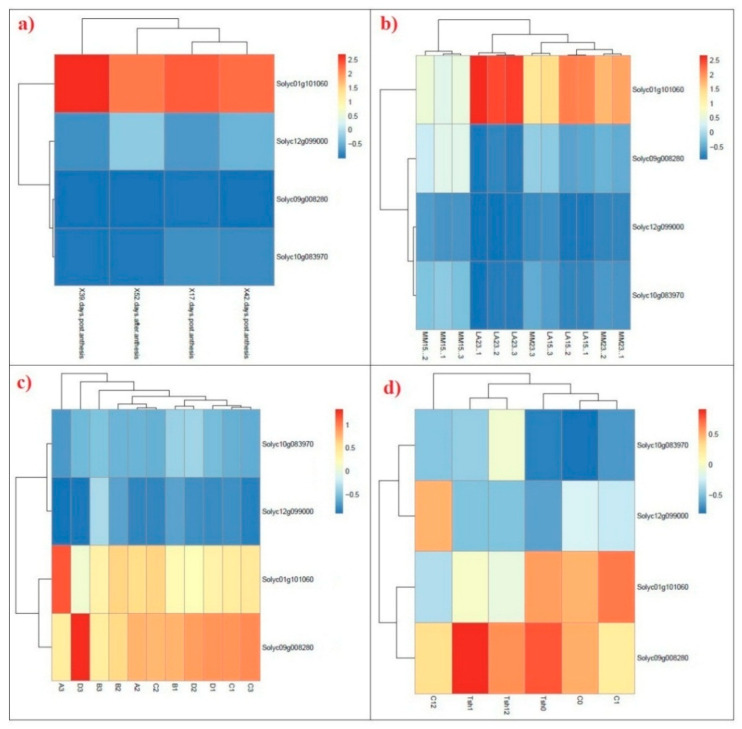
Heat map of the expression profiles of tomato SAMS genes under different stresses. (**a**) transcription profile of SAMS genes at developmental stages of tomato fruit obtained from RNA-seq dataset with accession ID: E-MTAB-4818 (X17, 39, 42, and 59 denote 17, 39, 42, and 52 days post-anthesis, respectively); (**b**) transcription profile of SAMS genes in tomato roots under low temperature stress obtained from RNA-seq dataset with accession ID: E-MTAB-4855 (LA23: *S. habrochaites* LA1777 at 23 °C; LA15: *S. habrochaites* LA1777 at 15 °C; MM23: *S. lycopersicum* Moneymaker at 23 °C; LA15: *S. lycopersicum* Moneymaker at 15 °C); (**c**) transcription profile of SAMS genes in tomato shoot under biotic stress [25], (A: control; B: treatment with *Pseudomonas syringae* pv. Tomato DC3000; C: nightly red light treatment; D: treatment with *P. syringae* pv. Tomato DC3000+ nightly red light treatment); (**d**) transcription profile of SAMS genes in tomato shoot under cold stress [26], (C0, C1 and C12: *S. lycopersicum* at 0, 1, and 12 h at 4 °C, respectively; Tsh0, Tsh1, and Tsh12: *S. habrochaites* at 0, 1, and 12 h at 4 °C, respectively).

To reveal the expression patterns of SAMS genes in different tissues and conditions, the available RNA-seq data of tomato species were analyzed. A heat map of tomato SAMS genes is shown in Figure 7. The expression patterns revealed that tomato SAMS genes were differentially expressed among tissues. For instance, Solyc01g101060 gene was especially expressed in fruit tissues (Figure 7a) and was strongly upregulated in the mature green fruit of tomato (at 39 days post anthesis). Among the tomato SAMS genes, the Solyc01g101060 gene showed the highest expression in root tissues, and it was downregulated in root tissues when the temperature was altered from 23 °C to 15 °C (Figure 7b). Furthermore, Solyc09g008280 showed higher expression in leaves than in other tissues (Figure 7c,d), while Solyc12g099000 showed weak expression in all analyzed samples. According to the RNA-seq results, Solyc09g008280 was more upregulated than were other genes under biotic stresses, such as treatment with *P. syringae* pv. Tomato DC3000 and nightly red light treatment (Figure 7c).

### 2.6. Expression Patterns of Tomato SAMS Genes Determined via qRT-PCR

**Figure 8:**
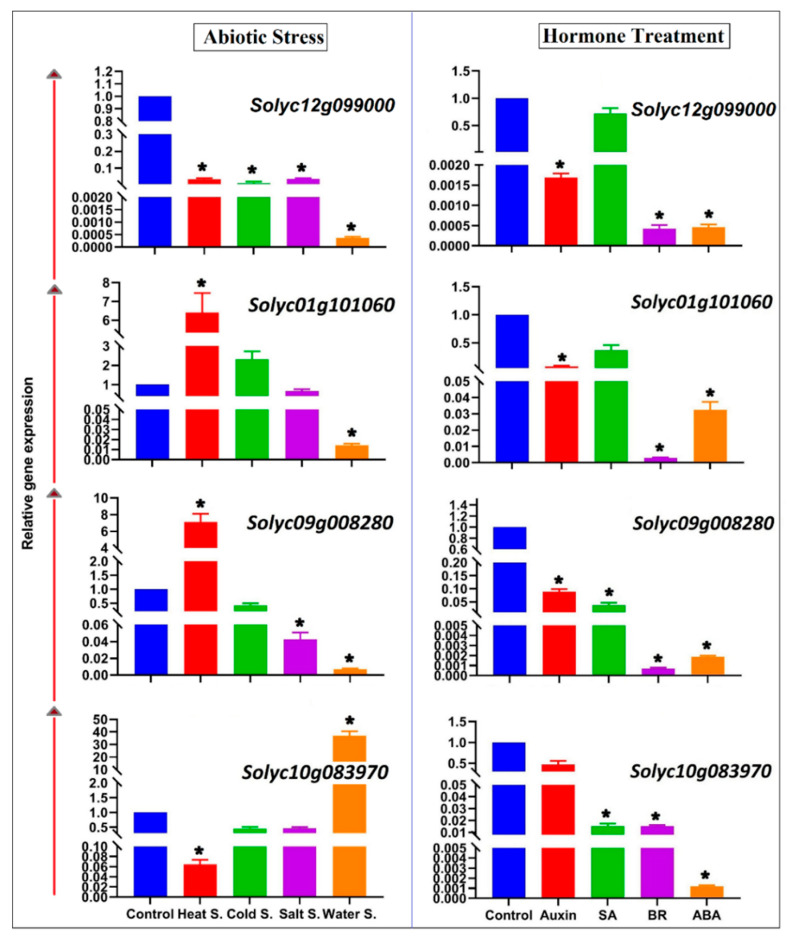
Expression patterns of SAMS genes under abiotic stress and hormone treatments. An asterisk above a bar indicates a significant difference between the experimental treatments and control treatment (according to Student’s *t*-test).

The expression patterns of members of a gene family can reveal aspects of their functions under different biological conditions. To elucidate the potential biological roles of tomato SAMS genes, their expression patterns were studied under four different abiotic stresses (salt, cold, heat, and water) and various exogenous hormone treatments (brassinosteroid (BR), salicylic acid (SA), auxin, abscisic acid (ABA)). qRT-PCR demonstrated that the SAMS genes were differentially expressed between each abiotic stress and control conditions (Figure 8). *Solyc12g099000* was downregulated under all abiotic stresses, especially under water stress. *Solyc01g101060* and *Solyc09g008280* expression was correlated with heat and water stress, which was downregulated under heat stress and strongly downregulated in response to water stress. In this study, the relative expression of *Solyc10g083970* was significantly increased in response to water stress, in contrast to that of the other studied tomato SAMS genes. Moreover, *Solyc01g101060* and *Solyc09g008280* showed similar expression patterns in response to abiotic stresses. In addition, the expression of tomato SAMS genes was induced in response to exogenous hormonal treatments (Figure 8). All tomato SAMS genes were strongly downregulated in response to exogenous BR application. Furthermore, a significant decrease was observed in the expression of *Solyc12g099000*, *Solyc09g008280*, and *Solyc10g083970* under ABA treatment. In addition, exogenous SA treatment did not affect *Solyc12g099000 and Solyc01g101060* expression but caused downregulation of *Solyc09g008280* and *Solyc10g083970.* Our results revealed that exogenous hormonal application reduced the expression of tomato SAMS genes.

## 3. Discussion

SAM is a key molecule involved in multiple cellular pathways, such as pathways related to ethylene and polyamine biosynthesis, methionine metabolism, and transmethylation and transsulfuration [4,5]. In the current study, SAM synthetase genes in different plant species were analyzed by an extensive use of bioinformatics tools. The SAMS genes in the selected plant species exhibited variation in GRAVY value, protein length, aliphatic index, isoelectric point, molecular weight, and instability index (Table 1). According to the instability index values, all of the studied SAMS proteins were stable. The instability index of a protein indicates the enzyme’s stability over reaction time [27]. In addition, the SAMS proteins showed high aliphatic index values, which confers high thermal stability and long half-lives to proteins [28]. Protein stability refers to the ability of a protein to retain function and structure in a specific condition. SAM is involved in several different pathways, and high stability preserves SAM structure in various cellular reactions. The predicted values of GRAVY, a solubility index [29], showed that the SAMS proteins of monocots, such as rice, sorghum, and barley, were less hydrophilic than the those of the studied dicot plants. In addition, cytosol and chloroplast were predicted as the subcellular localizations of SAMS proteins. SAM is localized in cytosol and can be transferred into chloroplast by specific carriers [30]. Methionine synthesis and S-adenosylmethionine (SAM) metabolism in plants can occur in chloroplast [30]. According to physiochemical results, it seems that SAMS proteins are thermostable proteins and can be employed in high-temperature reactions. 

According to the phylogenetic analysis, the motifs of group I were more conserved, indicating that dicot SAMS genes are probably derived from monocot SAMS genes. In particular, four conserved motifs found in all studied SAMS proteins are associated to general functions of these proteins. Besides, other conserved motifs observed in the same group reveal new insights on group-specific functions for SAMS proteins. In addition, the post-translational modifications of SAMS proteins were predicted based on phosphorylation and glycosylation sites. Phosphorylation and glycosylation processes are known as post-translational modifications in eukaryote organisms that play key roles in protein functions [27]. Phosphorylation plays key roles in signal transduction, protein–protein interactions and plant metabolism by modifying protein activities [31,32,33]. In the current study, the number of prediction phosphorylation sites ranged from 19 to 47, and three sunflower SAMS proteins (HanXRQChr07g0194741, HanXRQChr02g0051721 and HanXRQChr05g0148381) were identified as serine-rich proteins. Serine-rich proteins are highly phosphorylated, and most serine-rich proteins have pleiotropic effects on phenotypes and regulate various processes [34]. Phosphorylation can regulate several processes that affect features such as the stability and subcellular localization of proteins [27]. In this study, variation in the subcellular localization of serine rich-SAMS proteins was observed. Glycosylation can alter the molecular weight and stability of a protein [27,35]. Most proteins involved in the secretory pathway are extensively glycosylated in the endoplasmic reticulum [36]. According to glycosylation prediction results, most of the studied SAMS proteins were glycosylated in two conserved sites. These locations can be investigated in further studies related to functional genomics. The prediction results regarding miRNA targeting sites in transcript sequences of SAMS genes revealed that SAMS transcripts can be targeted by putative microRNAs. MicroRNAs or miRNAs compose a group of short, noncoding RNAs that have critical roles in many biological processes, including cell proliferation, development, organogenesis, and stress responses [37]. MiRNAs can regulate gene expression after transcription in collaboration with RNA-induced silencing complexes (RISCs) [38]. In this study, the target sites of gma-miR1520 and mtr-miR5275 in transcript sequences of soybean SAMS genes were predicted. Gma-miR1520 is involved in the responses to soybean cyst nematode infection and salt stress [39,40], while mtr-miR5275 is associated with arbuscular mycorrhizal symbiosis [41]. In addition, the target sites of osa-miR5528, osa-miR415, and hvu-miR6197 in transcript sequences of SAMS genes were observed. Osa-miR5528 has been identified as being involved in rice inflorescence at heading stage and in the response to drought stress [42], and osa-miR415 is involved in the response to rice stripe virus [43]. Furthermore, hvu-miR6197 is associated with the response to excess boron in barley [44]. MicroRNAs can alter the expression of target pre-mRNAs that are also regulated by several factors, such as biotic and abiotic stress. According to our results, SAMS genes can be regulated by different miRNAs in this complex (SAMS-miRNAs) to affect downstream pathways related to SAM.

**Figure 9:**
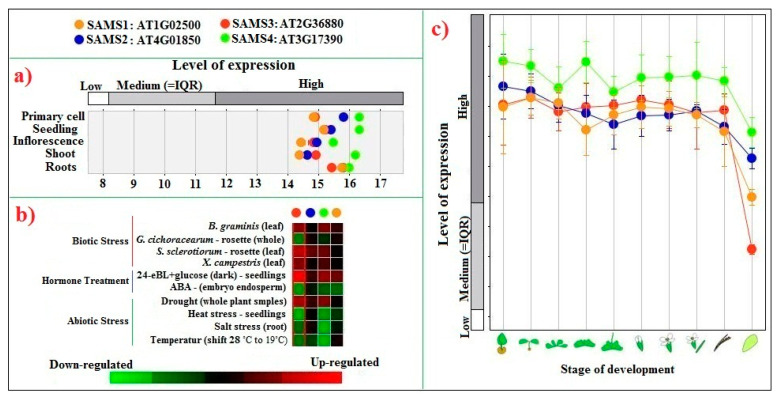
Expression levels of Arabidopsis SAMS genes in various tissues and organs (**a**) under different biotic and abiotic stresses and hormone treatments; (**b**) and at different development stages; (**c**) The data were obtained from Affymetrix Arabidopsis ATH1 genome array (10,615 samples) using Genevistigator [45].

The expression patterns of a gene family can reveal aspects of their functions under different biological conditions. To gain insight into the expression patterns of Arabidopsis SAMS genes, public microarray datasets [45] for different organs and stages of development and various abiotic and biotic stresses were analyzed (Figure 9). The results indicated that the expression patterns of SAMS genes varied among organs in Arabidopsis and tomato. Furthermore, SAMS genes showed differential expression from control expression in response to biotic and abiotic stresses. For example, *SAMS3* and *SAMS4* of Arabidopsis were upregulated under biotic stress and BR treatment and downregulated in response to abiotic stresses such as salt, heat and temperature stress and ABA application. The findings confirmed that BR can enhance pathogen resistance [46]. ABA is known as a hormone responsive to abiotic stresses such as drought, heat, low temperature, radiation and salt stress [47]. Besides, the transcript sequence of Arabidopsis-SAM3 is targeted by miR5021 (Table S2). Previous studies reported that miR5021 is involved in several pathways. For instance, miR5021 in *Mentha* spp. regulates genes that encode for enzymes involved in the essential oil biosynthesis [48]. In *Arabidopsis*, miR5021 negatively regulated genes involved in the abscisic acid signaling pathway and cell growth and indirectly linked with leaf senescence [49].

According to the expression patterns of tomato SAMS genes, the *Solyc01g101060* gene was especially expressed in fruit and root tissues, while *Solyc09g008280* gene was expressed primarily in leaves (Figure 7). These results revealed that tomato SAMS genes have tissue-specific expression patterns. Tissue-specific expression can provide insight into the different functions of target genes in different organs [50,51]. The qRT-PCR results revealed that *Solyc01g101060* and *Solyc09g008280* were downregulated under heat stress and water stress and that the expression of the Solyc10g083970 gene was downregulated under all abiotic stresses and hormone treatments (Figure 8). All studied tomato SAMS genes showed downregulation in response to salt stress. Wang et al. [52] similarly found that soybean SAMS genes are downregulated in root tissues in response to flooding and drought stresses. However, Sánchez-Aguayo et al. [53] reported that the expression of SAMS genes is increased in roots of tomato under salinity stress, and they observed a positive correlation between SAM activity and lignin deposition in the vascular tissues in response to salt stress. Similarly, Espartero et al. [21] found that the mRNA level of *SAMS1* is increased in response to NaCl stress in leaf tissue of tomato and that their transcript patterns can be maintained for at least 3 days. In addition, SAM enzyme is involved in glycine betaines biosynthesis as a key osmoprotectant under salt stress [54]. In this study, tomato SAMS genes showed differential expression in response to different stresses. Tomato SAMS genes were investigated under hormone treatments, and their expression patterns were found to be downregulated in response to exogenous hormone treatments. In particular, BR strongly decreased the expression pattern of SAMS genes (Figure 8). Phytohormones such as BR and auxin can reduce ethylene biosynthesis in Arabidopsis by interacting with SAM synthases, and ethylene biosynthesis has been shown to decrease in double mutant lines of SAMS1 and SAMS2 (*mat1* and *mat2*) [23]. Both auxin and BR may negatively control the SAMS genes (SAMS1 and SAMS2 of Arabidopsis) from FERONIA (FER) as a receptor-like kinase of plasma membrane [23]. It is also known that exogenous application of BR decreases the free ACC and ethylene content in root and shoot tissue but maintains the polyamines level in lettuce plants under salt stress [55]. In the present study, the expression of *Solyc12g099000*, *Solyc09g008280*, and *Solyc10g083970* was significantly decreased under ABA treatment. SAMS genes have multiple biological roles, including participation in methionine metabolic pathways [4], and they have crucial roles in ethylene and polyamine biosynthesis and plant cell signaling [52,56]. However, ethylene and polyamine have antagonistic effects to each other. Polyamines can reduce the ethylene biosynthesis by controlling expression of ACC synthase (ACS) and ethylene can inhibit the activity of enzymes involved in polyamine biosynthesis such as SAMDC [57]. The enzyme ACS that catalyzes ACC from SAM (for details see Figure 1) is introduced as a regulatory crosstalk point between ethylene biosynthesis and polyamine pathway [15]. Moreover, this enzyme is encoded by multi ACS genes that have differential expression in response to distinct inducers affecting the conversion of SAM to ACC in ethylene biosynthesis [15]. The overexpression of SAMS in tobacco was found to induce the expression levels of SAMDC under cold stress while ACS and ACO are not affected [58,59]. In alfalfa, the expression of SAM1 gene was found associated with polyamines in response to cold stress [58], suggesting a positive correlation between SAM activity and SAMDC under abiotic stress. Our results are consistent with previous findings as this study reveals that SAMS genes are involved in hormone signaling and that ABA and BR can negatively regulate downstream pathways related to SAMS genes.

## 4. Materials and Methods 

### 4.1. Identification and Sequence Analysis of SAMS Genes

Protein sequences of SAMS genes in *Arabidopsis thaliana* were obtained from the Arabidopsis Information Resource (TAIR10) database (ftp://ftp.arabidopsis.org). The protein sequences of Arabidopsis-SAMS genes were used as queries in a BLASTp analysis against the proteomes of tomato, eggplant, sunflower, barley, rice, *Triticum urartu*, sorghum, *Medicago truncatula* and soybean to identify SAMS proteins. To assess the presence of the protein domain S-adenosylmethionine synthetase, all predicted-SAMS proteins were analyzed by Conserved Domain Database (https://www.ncbi.nlm.nih.gov/Structure/cdd/cdd.shtml). The biochemical properties of selected proteins, including protein length, molecular weight (MW), isoelectric point (pI), instability index, aliphatic index and the grand average of hydropathy (GRAVY), were predicted by ExPASy ProtParam [60] (https://web.expasy.org/protparam/). In addition, the subcellular localization of SAMS genes was predicted using the BUSCA webserver [61].

### 4.2. Evolutionary Analysis

The amino acid sequences of all predicted SAMS proteins from the ten species were aligned by ClustalX, and a phylogenetic tree was constructed using the neighbor-joining method of Clustal Omega (https://www.ebi.ac.uk/Tools/msa/clustalo/). The phylogenetic tree was visualized using Evolview v3 server [62].

### 4.3. Conserved Motif Recognition

To identify the conserved motifs, the amino acid sequences of SAMS proteins were analyzed using the MEME v4.12.0 server (http://meme-suite.org/tools/meme) with the following parameters: motif width, between 6 and 50 residues; maximum number of motifs, 10 [63].

### 4.4. Prediction of Post-Translational and Post-Transcriptional Modification Sites

The phosphorylation sites among amino acid sequences of SAMS genes were predicted by the NetPhos 3.1 server (http://www.cbs.dtu.dk/services/NetPhos/) [64]. The N-glycosylation sites of SAMS proteins were predicted using the NetNGlyc 1.0 server (http://www.cbs.dtu.dk/services/NetNGlyc/) [65]. The threshold in NetPhos 3.1 and NetNGlyc 1.0 servers was set to a potential value >0.5. To predict the target sites of microRNA, the transcript sequences of selected SAMS genes were run against all published plant microRNAs using the psRNATarget server (http://plantgrn.noble.org/psRNATarget/) at an expectation level ≤2.5 [66].

### 4.5. Expression Profile Analysis Using RNA-Seq and Microarray Data

For the expression profiling of tomato SAMS genes, we utilized the 33 samples of published RNA-seq datasets that included transcription profiles of tomato fruit at four developmental stages (accession ID: E-MTAB-4818), root transcriptome profiles of chilling-sensitive tomato (*S. lycopersicum* cv. Moneymaker) and the cold-tolerant wild tomato (*S. habrochaites* LA1777) at optimal and suboptimal temperature (accession ID: E-MTAB-4855), transcriptome profiles of tomato shoot under biotic stress (*Pseudomonas syringae* pv. tomato DC3000) and red light treatment (accession GSE64087 of NCBI Sequence Read Archive database) [25], and low-temperature transcriptomes of *S. lycopersicum* and *S. habrochaites* [26]. The expression levels of tomato SAMS genes were normalized ((x-µ)/sd) based on the published expression units, and a heatmap was constructed with the ‘*pheatmap*’ package of the R language. In addition, expression profiles of SAMS genes in *Arabidopsis thaliana* under different conditions and development stages were obtained from Affymetrix Arabidopsis ATH1 genome array (10,615 samples) using Genevistigator [45].

### 4.6. Plant Growth and Treatments

Seeds of *Solanum lycopersicum* cv. Moneymaker were sown in plates containing 60% vermicompost and 40% perlite and maintained under a 16 h photoperiod (5000 Lux) and a 24 ± 2 °C day/night temperature. After five weeks, healthy tomato seedlings were treated with different hormones and abiotic stresses. Treatments included brassinosteroid (BR) (5 µM of epi-brassinosteroid), salicylic acid (SA) (30 mg/L), auxin (1 mg/L), abscisic acid (ABA) (10 mg/L), salt stress (150 mM of NaCl), cold stress (24 h at 8 °C), heat stress (24 h at 40 °C) and water stress. For the hormone treatments (BR, SA, ABA, and auxin), each hormone solution was sprayed onto the tomato seedlings at two times. After 6 h, whole shoots from each treatment were separately harvested and stored in liquid nitrogen at −80 °C. For the salt stress treatment, tomato seedlings were irrigated with a solution of 150 mM NaCl for two days. Whole shoots were harvested at the end of treatment. Cold and heat stresses were imposed by maintaining seedlings at 8 °C and 40 °C, respectively, for 24 h, and then the whole shoots were harvested. For the water stress treatment, irrigation was withheld for 10 days; whole shoots were then collected. Seedlings that had been maintained at 24 ± 2 °C were harvested as control samples. After applying the treatments, all collected samples of each treatment were immediately placed in liquid nitrogen and stored at −80 °C. All experimental treatments included three biological replicates.

### 4.7. RNA Extraction and qRT-PCR Analysis

The total RNA from the frozen samples was extracted using the RNX TM-Plus kit (Sinaclon, Iran) according to the manufacturer′s recommendations. The quantity and quality of the extracted RNA were analyzed using a Nano Photometer (Implen N50) and agarose gel electrophoresis. First-strand complementary DNA (cDNA) was synthesized using 1 µg total RNA (treated with RNase-free DNase I (Thermo Fisher Scientific)) and reverse transcriptase (Roche, Germany) according to the manufacturers′ protocols. The specific primers for the four tomato SAMS genes (Solyc12g099000, Solyc01g101060, Solyc09g008280, and Solyc10g083970) and EF-1-α (Solyc06g005060), as a reference gene, were designed using Primer3 Plus online software (Table S1). Real-time PCR was performed in 10 µl volumes using the Applied Biosystems StepOne^TM^ system and RealQ Plus 2x Master Mix Green high ROX^TM^ (Ampliqon) according to the instructions of the manufacturers. The real-time PCR conditions were as follows: 95 °C for 10 min, followed by 35 cycles at 95 °C for 15 s and 60 °C for 20 s. The melting curve for each sample was obtained after 35 cycles at the temperature range from 60 to 95 °C. The relative expression patterns of SAMS genes were calculated using the 2-ΔΔCt method [67]. All graphs were constructed using Graphpad Prism 5.0 software based on the mean and standard division (SD) of the expression values of each gene.

## 5. Conclusions

In this study, members of the SAMS gene family were evaluated in several plant species, with focus on physiochemical properties, post-transcriptional and translational modifications, and expression patterns. The SAMS proteins varied in protein characterization features, conserved motifs, phylogenetic clustering analysis, post-translation modifications, and gene expression patterns. The tomato SAMS genes showed differential expression in response to stresses and hormone treatments. The various expression patterns of SAMS genes indicated that these genes play critical regulatory roles in metabolic pathways involved in the responses to environmental stresses. In addition, it seems that ABA and BR inhibit the ethylene and polyamines biosynthesis induced by SAMS genes. Our study provides very useful outputs and novel insights into the main roles of the SAMS gene family in plants. The overall results will be useful for future studies on the molecular functions of SAMS genes.

## Figures and Tables

**Table 1:** The properties of SAMS genes in the studied plants.

Organism	Locus ID	Length (aa)	MW (KDa)	pl	Instability Index	Aliphatic Index	GRAVY	Subcellular Localization
Arabidopsis	AT2G36880	390	42.497	5.76	stable	83.74	−0.236	Cytoplasm
	AT4G01850	393	43.255	5.67	stable	79.34	−0.353	Cytoplasm
	AT3G17390	393	42.796	5.51	stable	85.04	−0.255	Cytoplasm
	AT1G02500	393	43.158	5.51	stable	80.33	−0.306	Cytoplasm
Tomato	Solyc01g101060.3	393	43.301	5.51	stable	82.80	−0.335	Cytoplasm
	Solyc09g008280.2	390	42.652	5.76	stable	82.95	−0.283	Cytoplasm
	Solyc10g083970.1	390	42.660	6.12	stable	80.97	−0.313	Cytoplasm
	Solyc12g099000.2	393	43.082	5.41	stable	84.05	−0.313	Cytoplasm
Eggplant	Sme2.5_00004.1_g00028.1	390	42.624	5.76	stable	81.97	−0.290	Cytoplasm
	Sme2.5_00187.1_g00013.1	393	43.122	5.68	stable	84.30	−0.307	Cytoplasm
	Sme2.5_07249.1_g00002.1	394	43.367	5.49	stable	82.34	−0.331	Chloroplast
	Sme2.5_07301.1_g00003.1	390	42.627	6.08	stable	81.23	−0.305	Cytoplasm
Sunflower	HanXRQChr01g0027791	238	26.223	9.13	stable	92.52	−0.153	Cytoplasm
	HanXRQChr01g0027801	390	42.969	5.97	stable	81.97	−0.346	Cytoplasm
	HanXRQChr02g0051721	457	49.894	5.93	stable	84.03	−0.227	Chloroplast
	HanXRQChr05g0148381	455	49.847	5.42	stable	83.76	−0.257	Chloroplast
	HanXRQChr07g0194741	453	49.887	5.97	stable	83.05	−0.237	Chloroplast
	HanXRQChr13g0400841	437	48.132	6.22	stable	83.62	−0.291	Chloroplast
	HanXRQChr14g0454811	341	37.276	6.42	stable	84.34	−0.297	Cytoplasm
*Triticum urartu*	XP_020178978.1	394	42.765	5.52	stable	85.86	−0.203	Cytoplasm
	EMS55466.1	394	42.788	5.61	stable	84.39	−0.212	Cytoplasm
	EMS52834.1	479	52.800	6.51	stable	81.98	−0.290	Chloroplast
Barley	HORVU6Hr1G034650	396	43.158	5.33	stable	81.72	−0.253	Cytoplasm
	HORVU6Hr1G063490	394	42.766	5.52	stable	85.86	−0.203	Chloroplast
	HORVU6Hr1G063540	394	42.814	5.49	stable	83.40	−0.219	Cytoplasm
	HORVU6Hr1G063560	394	42.828	5.58	stable	83.40	−0.220	Cytoplasm
Rice	LOC_Os01g22010	394	42.901	5.68	stable	81.14	−0.264	Cytoplasm
	LOC_Os01g18860	396	43.310	5.22	stable	82.70	−0.274	Cytoplasm
	LOC_Os05g04510	396	43.220	5.74	stable	82.45	−0.289	Cytoplasm
Sorghum	Sobic.003G140000	396	43.257	5.32	stable	83.69	−0.284	Cytoplasm
	Sobic.003G151600	394	42.785	5.50	stable	82.13	−0.237	Cytoplasm
	Sobic.009G033600	396	42.976	5.57	stable	83.21	−0.260	Cytoplasm
*Medicago truncatula*	Medtr1g063060	408	44.904	6.24	stable	81.67	−0.294	Cytoplasm
	Medtr2g046710	396	43.174	5.77	stable	83.43	−0.287	Cytoplasm
	Medtr4g123810	393	42.916	5.67	stable	85.29	−0.254	Cytoplasm
	Medtr7g102120	390	42.654	6.36	stable	82.46	−0.300	Cytoplasm
	Medtr7g110310	394	43.279	5.59	stable	80.86	−0.327	Cytoplasm
Soybean	Glyma.07G233800	392	43.055	5.65	stable	82.53	−0.330	Cytoplasm
	Glyma.10G144300	389	42.620	5.41	stable	85.42	−0.259	Cytoplasm
	Glyma.17G039000	392	42.981	5.43	stable	83.52	−0.328	Cytoplasm
	Glyma.03G184000	390	42.727	6.13	stable	81.46	−0.298	Cytoplasm
	Glyma.03G223000	394	43.224	5.57	stable	80.13	−0.337	Cytoplasm
	Glyma.19G220200	394	43.250	5.57	stable	79.87	−0.345	Cytoplasm
	Glyma.10G054500	390	42.608	5.86	stable	82.46	−0.283	Cytoplasm
	Glyma.13G141600	390	42.586	5.82	stable	82.46	−0.276	Cytoplasm
	Glyma.15G190500	395	43.053	5.50	stable	82.89	−0.304	Cytoplasm

## Supplementary Materials

The following are available online at https://www.mdpi.com/2223-7747/9/5/586/s1, Table S1: List of primers used for real-time PCR, Table S2: Putative miRNAs targeting the transcripts of SAMS genes at an expectation level < 4.00.
