# Supplementary material for: Insights into the SAM Synthetase Gene Family and Its Roles in Tomato Seedlings under Abiotic Stresses and Hormone Treatments
Source: Plants (Basel). 2020 May 4;9(5):586. doi: 10.3390/plants9050586 (PMC7284622; doi:10.3390/plants9050586)
Supplement: Supplementary file 1 [file plants-09-00586-s001.pdf]

Table S1- List of used primers in real-time PCR

Gene ID	Name	Primer (5′-3′)	Product size (bp)
Solyc06g005060	*EF-1-α*	Forward: *CGTGGTTATGTTGCCTCAAA*<br>Reverse: *ACAGCAATGTGGGAAGTGTG*	146
Solyc12g099000	*SAMS*	Forward: *CATGTTTGGCTATGCCACTG*<br>Reverse: *GCCAAGAGCAAGTTCCATTC*	116
Solyc01g101060	*SAMS*	Forward: *TGACAACGGTGCTATGGTTC*<br>Reverse: *CTTGATGACACGCTCTTTGTG*	115
Solyc09g008280	*SAMS*	Forward: *AGATCACAACCAAGGCCAAG*<br>Reverse: *AGGTGAAGTGAAGCCAATGC*	77
Solyc10g083970	*SAMS*	Forward: *CATGTTTGGCTATGCCACTG*<br>Reverse: *GCCAAGAGCAAGTTCCATTC*	116

Table S2- Putative miRNAs targeted the transcripts of SAMS genes at expectation level < 4.00

miRNA ID	Target ID	miRNA sequence (3-5)	Target position	Expectation	Inhibition
ath-miR843	AT1G02500	AGGUUACUUCGAGCUGGAUUU	1333-1353	3.0	cleavage
ath-miR5021	AT2G36880	AAAAGAAGAAGAAGAAGAGU	1556-1575	3.5	cleavage
hvu-miR6197	HORVU6Hr1G063490	GCAGAAUGUAAAUCCUUGUCU	837-857	2.5	cleavage
osa-miR1858	LOC_Os01g22010	CGGGGUGAGGCAGGAGGAGAG	567-587	3.0	cleavage
osa-miR1858	LOC_Os05g04510	CGGGGUGAGGCAGGAGGAGAG	570-590	3.0	cleavage
osa-miR2055	LOC_Os01g22010	CUUUGGUGGAAGGGUUCCUUU	1296-1316	3.5	cleavage
osa-miR5528	LOC_Os01g18860	CCGUUGUAGAUUUUGGCAGAA	1330-1350	2.5	cleavage
osa-miR2874	LOC_Os05g04510	CUGUGACAAACUGUGACAAGU<br>GUA	1758-1781	3.5	cleavage
mtr-miR2119	Medtr1g063060	GAUGAGGUGUGGAGGGAAACU	11-31	3.5	cleavage
gma-miR2606	Glyma.07G233800	AUGGCAAGGAAUUCACGAAAA	111-131	3.5	cleavage
gma-miR9726	Glyma.03G223000	CUUCUUUUUUUAUUACGGAUA<br>U	1195-1216	3.0	cleavage
gma-miR1529	Glyma.19G220200	AUUGCUAAUUAACAAAGGAA<br>AUU	1801-1823	2.5	cleavage
gma-miR9726	Glyma.19G220200	CUUCUUUUUUUAUUACGGAUA<br>U	1173-1194	3.0	cleavage
gma-miR9723	Glyma.19G220200	CUCAACAGGUUUAGAGGAAAC	1246-1266	3.5	cleavage
gma-miR9748	Glyma.15G190500	CAGUAGGGAGAUGUGAAGGA<br>AG	1503-1524	3.5	cleavage
